# Cryo-PRO facilitates whole blood cryopreservation for single-cell RNA sequencing of immune cells from clinical samples

**DOI:** 10.1101/2024.09.18.24313760

**Published:** 2024-09-19

**Authors:** Alyssa K. DuBois, Pierre O. Ankomah, Alexis C. Campbell, Renee Hua, Olivia K. Nelson, Christopher A. Zeuthen, M. Kartik Das, Shira Mann, Abigail Mauermann, Blair A. Parry, Nathan I. Shapiro, Michael R. Filbin, Roby P. Bhattacharyya

**Affiliations:** 1 Broad Institute, Cambridge MA, USA; 2 Massachusetts General Hospital, Boston MA, USA; 3 Beth Israel Deaconess Medical Center, Boston MA, USA

## Abstract

Single-cell RNA sequencing (scRNA-seq) of peripheral blood mononuclear cells (PBMCs) has enhanced our understanding of host immune mechanisms in small cohorts, particularly in diseases with complex and heterogeneous immune responses such as sepsis. However, PBMC isolation from blood requires technical expertise, training, and approximately two hours of onsite processing using Ficoll density gradient separation (‘Ficoll’) for scRNA-seq compatibility, precluding large-scale sample collection at most clinical sites. To minimize onsite processing, we developed Cryo-PRO (Cryopreservation with PBMC Recovery Offsite), a method of PBMC isolation from cryopreserved whole blood that allows immediate onsite sample cryopreservation and subsequent PBMC isolation in a central laboratory prior to sequencing. We compared scRNA-seq results from samples processed using Cryo-PRO versus standard onsite Ficoll separation in 23 patients with sepsis. Critical scRNA-seq outputs including cell substate fractions and marker genes were similar for each method across multiple cell types and substates, including an important monocyte substate enriched in patients with sepsis (Pearson correlation 0.78, p<0.001; 70% of top marker genes shared). Cryo-PRO reduced onsite sample processing time from >2 hours to <15 minutes and was reproducible across two enrollment sites, thus demonstrating potential for expanding scRNA-seq in multicenter studies of sepsis and other diseases.

## INTRODUCTION

Single-cell RNA sequencing (scRNA-seq) is a pivotal methodology with great potential for advancing the understanding of biological systems (1). It has revealed previously unrecognized cell types and transcriptional substates within complex tissues and demonstrated differences in gene expression that illuminate pathophysiological variation in many aspects of human disease (2-4). ScRNA-seq has particular appeal for dissecting the cellular basis for heterogeneity in diseases and opening pathways to precision medicine. Sepsis, for example, is a syndrome with expansive differences in clinical course and outcome between patients that is impacted substantially by heterogeneity in patients’ immunological responses. However, there has been limited progress in understanding this variation using existing methods. In particular, transcriptional profiling in patients with sepsis has mostly relied on bulk RNA sequencing (bulk RNA-seq) to generate averaged signatures from all circulating immune cells, obscuring the cellular basis and underlying mechanisms of immune dysfunction (5-9).

In previous work, our group has used scRNA-seq to profile circulating peripheral blood mononuclear cells (PBMCs) in urosepsis, identifying a unique CD14+ monocyte subtype (monocyte substate 1, or MS1), that is expanded in sepsis relative to infection without sepsis (10). These monocytes have a gene expression profile similar to myeloid-derived immune suppressor cells, which are immune regulatory cells that inhibit T cell activation, proliferation and cytotoxic activity (11). MS1 cells may therefore play an immunosuppressive role in sepsis and contribute to an important transcriptional subphenotype, or “endotype”, of sepsis (12). Several other studies have employed scRNA-seq in small sepsis cohorts (13-16). However, resolution of sepsis endotypes and the contributory roles of immune cell subtypes requires large cohorts enrolled at multiple, geographically-separated clinical sites. Such large-scale studies are necessary to appropriately represent the heterogeneity of sepsis with its variable pathogen types, anatomic sites of infection, timing of presentation, severity, trajectory, clinical characteristics (e.g., age, sex, race and ethnicity, comorbidities), and complex host immune responses. Leveraging the resolution of scRNA-seq at a scale that allows sufficient sampling of all relevant subphenotypes of sepsis has the potential to enable endotyping assessment with sufficient accuracy to impact sepsis care.

Implementing scRNA-seq studies in clinical settings at scale is challenged by several logistical barriers. Blood, which offers a diverse and dynamic snapshot of the systemic response to infection, serves as a key resource for investigating immune responses in sepsis and other conditions (17). However, since live blood cells are highly sensitive to environmental perturbations, it is necessary to either process samples rapidly before sequencing or employ cryostorage for later analysis. These steps help minimize any transcriptional changes in cells caused by stimuli after blood collection. Therefore, processing the blood sample to a point where transcription is halted (e.g., by freezing live cells or fixing them unless sequencing is performed immediately) often falls to operators at the sample collection site. Currently, standard practice for scRNA-seq studies of PBMCs involves a density gradient centrifugation step immediately following blood draw (Ficoll-paque processing, or "Ficoll") to isolate and store immune cells (18). This process, which must be completed promptly at the site of blood collection, is resource-intensive, time-consuming, sensitive to protocol variations, and requires technical skill and training. The complexity of real-time processing of whole blood samples has limited the widespread use of scRNA-seq in clinical investigations. Moreover, the lack of standardization in processing and analysis can lead to batch effects, hindering comparisons across sites and between studies.

To overcome the practical limitations of scRNA-seq in clinical settings, we developed Cryo-PRO (Cryopreservation with PBMC Recovery Offsite), a method for isolating PBMCs from cryopreserved whole blood samples. The approach utilizes magnetic depletion of red blood cells followed by fluorescence-activated cell sorting to recover immune cells for scRNA-seq. Cryo-PRO enables the immediate cryopreservation of whole blood samples at clinical sites, with onsite freezing and storage, allowing for their transfer at a later time to a centralized laboratory for PBMC isolation and scRNA-seq. In this study, we directly compare the scRNA-seq output from sepsis patient samples processed using Cryo-PRO with those processed by the standard onsite Ficoll-gradient separation method. Our findings demonstrate technical equivalence and biological reproducibility between the two methods. Cryo-PRO has the potential to enable broad application of scRNA-seq to multicenter studies and clinical trials by simplifying sample collection and centralizing cell isolation to improve cost efficiency, minimize batch effects, and increase sample sizes. This method could advance our understanding of the complexity of sepsis and other heterogeneous diseases, enabling development of precision diagnostics and targeted therapeutic strategies.

## RESULTS

Patients greater than 18 years of age who presented to the Emergency Departments of two large, academic hospitals in Boston MA, Massachusetts General Hospital (MGH) and Beth Israel Deaconess Medical Center (BIDMC), with clinical concern for sepsis or septic shock with associated organ dysfunction were enrolled in the study. Up to 10 mL of blood was obtained from patients and processing was initiated onsite using two methods: 1) standard Ficoll gradient separation from whole blood by following standard procedures for isolating and freezing PBMCs (18), followed by −80°C freezing; and 2) Cryo-PRO, by adding dimethyl sulfoxide (DMSO) to a final volume of 10% in 1 mL aliquots of fresh whole blood and immediately freezing at −80°C. To enable comparison of outcomes by processing site, for a subset of patients, up to 20 mL of blood (separated in two 10-mL tubes) was obtained; one tube was immediately couriered to the other clinical site while one tube remained at the enrolling site. Processing at both sites using both Ficoll and Cryo-PRO began at the same time upon sample receipt at the receiving site. Blood from one healthy donor was obtained from Research Blood Components (Watertown, MA) and processed using both Ficoll and Cryo-PRO methods to use as a reference standard. All samples were sent to the Eli and Edythe L. Broad Institute of MIT and Harvard (Broad Institute) for long term storage at −140°C and subsequent processing and scRNA-seq.

We adjudicated 23 subjects based on the presence of sepsis or bacterial infection for sample processing and sequencing. Septic shock requiring vasopressors for over 24 hours was present in 15 subjects (65%), sepsis without shock in 6 subjects (26%), and bacterial infection not meeting Sepsis-3 criteria (19) in 2 subjects (9%). Bacteremia was present in 7 (30%) of the 23 subjects. The median patient age was 66 years (IQR 62.5 - 76.5), with 35% women. The healthy donor was a 63 year old man.

**Figure 1.**
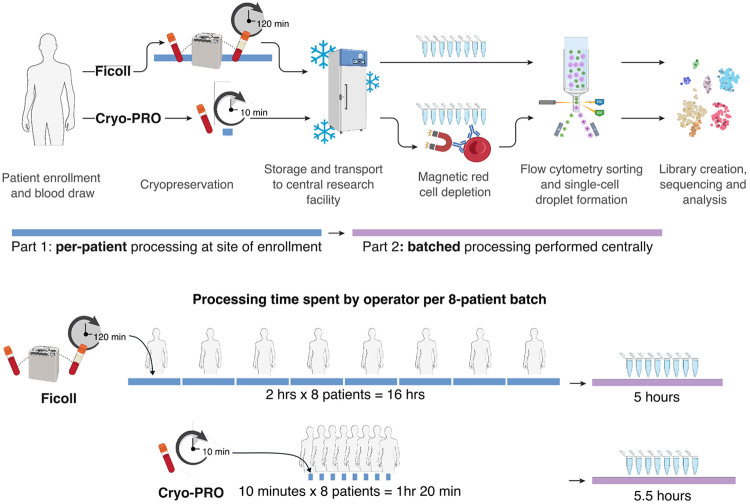
Overview of sample processing methods; Ficoll and Cryo-PRO. Cryo-PRO is designed to expedite sample processing at the site of collection by incorporating a whole blood cryopreservation step (and subsequent red cell depletion step) to replace standard Ficoll processing. Blue bars represent processing done at site of enrollment; purple bars represent processing done at centralized laboratory and include steps up to single-cell droplet formation.

Of the 23 included subjects, 8 were processed in parallel at both sites and 15 were processed only at the site of enrollment. Patient-paired frozen Ficoll and Cryo-PRO samples were processed for scRNA-seq at the Broad Institute. Processing included a magnetic red blood cell depletion step (Cryo-PRO samples only), fluorescence-activated cell sorting to recover DAPI− CD45+ CD235a− CD15− cells, and a standard workflow for droplet-based single-cell RNA capture with surface proteome measurement (10X Genomics Chromium Next GEM 5’ V2 Kit with cellular indexing of transcriptional epitope sequencing (CITE-seq)) (see Methods) (20). Sample hashing was used to enable pooling of eight samples per processing batch, and to facilitate post-sequencing demultiplexing and multiplet detection. An overview of the sample collection, storage, and processing strategies is summarized in Figure 1.

### Samples processed using Cryo-PRO yield high quality scRNA-seq data with minimal on-site processing time

**Figure 2.**
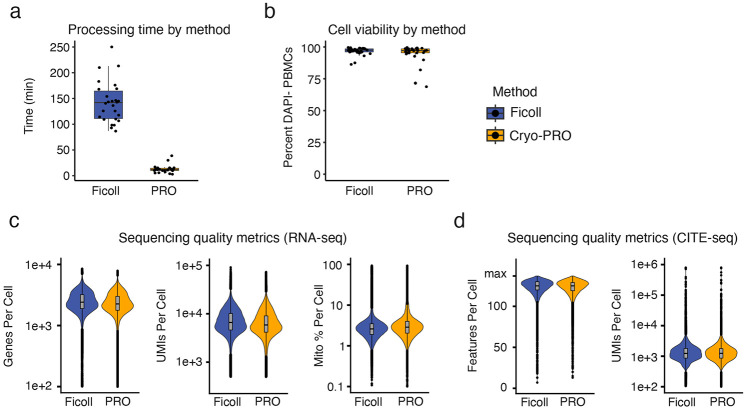
Processing time and quality metrics by cryopreservation method. **(a)** “Hands-on” time spent by operators at clinical sites to process patient samples from initiation of processing after blood draw to placing the sample in a freezer for storage. **(b)** Percent of CD45+ CD235a− CD15− cells staining DAPI negative on flow cytometry as an indicator of cell membrane integrity and cell viability. **(c)** Violin plots of RNA sequencing quality metrics by method (left to right): unique genes per cell, unique molecular identifiers (UMIs) of RNA transcripts per cell, percent of transcripts represented by mitochondrial genes per cell. **(d)** Violin plots of CITE-seq quality metrics by method: unique surface protein features (left panel) and UMIs (right panel) per cell (detected via CITE-seq). PRO denotes Cryo-PRO.

The mean time required for complete on-site processing (from processing start time to storage at −80°C) for Ficoll samples was 2 hours and 23 minutes (SD: 40 minutes), while Cryo-PRO samples required an average of 13 minutes (SD: 7 minutes) (Figure 2a). The proportion of viable PBMCs recovered by either method was estimated using live/dead staining during flow cytometry sorting. The mean proportion of live (DAPI−) PBMCs (CD235a− CD15− CD45+ cells) was 96.7% (SD 3.0%) for Ficoll samples and 94.1% (SD 8.4%) for Cryo-PRO samples (Figure 2b).

The Cellranger pipeline (10X Genomics) was used to process the raw sequencing data, and the Seurat V5 package in R (21) was used for subsequent analysis of single-cell sequencing data (see Methods). Multiplets (cells associated with more than one patient hashtag) were removed from analysis. We recovered an average of 2,690 (SD 950) and 2,472 (SD 918) singlet cells per sample for the Ficoll and Cryo-PRO methods respectively (Supplemental Figure 1). Sequencing quality was assessed using the following standard metrics: 1) number of genes sequenced per cell, 2) number of unique molecular identifiers (UMIs) per cell, and 3) percent of mitochondrial genes sequenced per cell. Higher numbers of genes per cell and UMIs per cell indicate greater per-cell transcript recovery, while a greater percentage of mitochondrial genes suggests cell damage (22). Quality metrics showed similar distributions between methods (Figure 2c). The majority of cells (97.6% from Ficoll processing and 94.4% from Cryo-PRO) passed commonly-used quality thresholds (i.e., >250 genes per cell, >1,000 UMIs, and <10% mitochondrial genes) (23). Detection of antibody-derived tags (ADT) used for CITE-seq surface proteome measurement was similar between the two methods (Figure 2d). Quality metrics were similar between methods at an individual patient level (Supplemental Figure 2).

### Cryo-PRO enables identification of immune cell transcriptional substates and gene expression patterns

**Figure 3.**
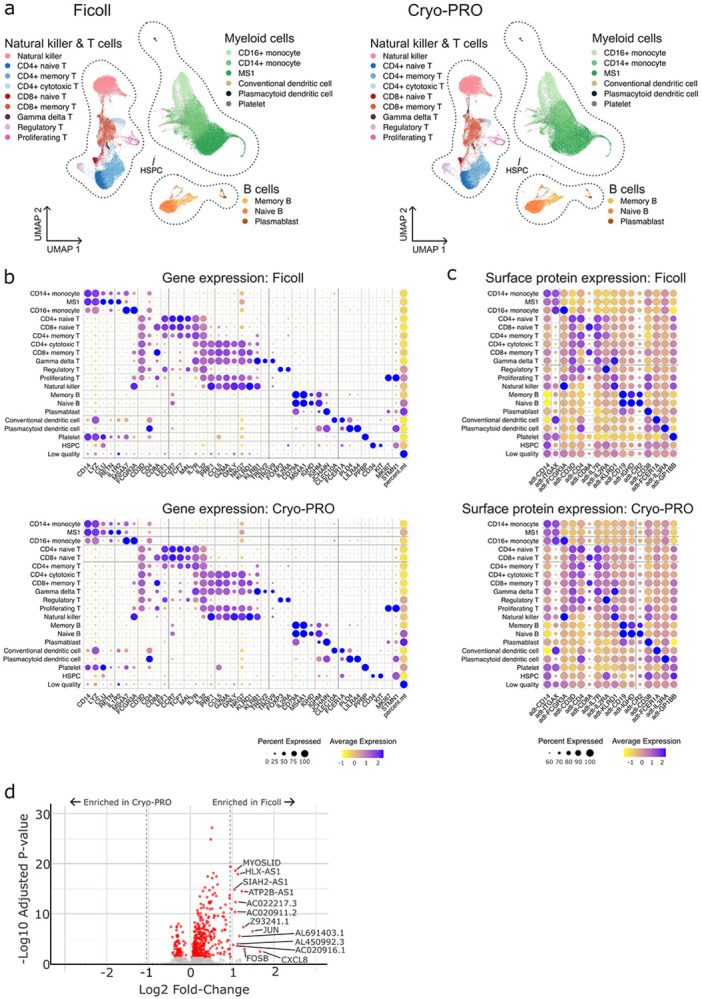
Comparison of gene and protein profiling by method. **(a)** Two-dimensional uniform manifold approximation and projection (UMAP) of cells by processing method; Ficoll (left) and Cryo-PRO (right). Dotted outlines represent major PBMC lineages. Cell substates were identified by clustering cells of each method independently; substate identities were then projected onto a shared set of UMAP axes (see Methods). **(b)** Dot plots of key marker genes and percent mitochondrial reads (percent.mt) for cell substates identified in scRNA-seq analysis by method (top: Ficoll, bottom: Cryo-PRO). **(c)** Dot plots of key surface marker proteins (detected using CITE-seq) for each cell substate. For both (b) and (c), color represents scaled relative expression (blue = higher expression), and size represents the proportion of cells in each substate where the feature was detected. **(d)** Volcano plot showing genes differentially up-regulated (positive log_2_FC) or down-regulated (negative log_2_FC) in Ficoll compared to Cryo-PRO using pseudobulk expression data from all cells (see Methods). Genes with adjusted p-values of less than 0.05 are shown in red; those with p < 0.05 and abs(log_2_FC) > 1 are labeled.

We next assessed whether Cryo-PRO generates scRNA-seq datasets of sufficient quality to reproduce biologically relevant results compared to Ficoll. ScRNA-seq analysis was performed separately for cells obtained from each processing method (86,083 cells for Ficoll and 79,089 cells for Cryo-PRO) to ensure independent identification of cell identity and gene expression patterns (see Methods). Clusters of dead and dying cells, identified by the predominance of mitochondrial genes, were removed from further analysis as an additional quality control measure (24). We identified transcriptionally similar cells that expressed canonical marker genes for the major mononuclear immune cell lineages (i.e., T cells, B cells, natural killer cells, monocytes, and dendritic cells). Subclustering within each cell type identified higher-resolution clusters of cells with additional transcriptional similarity (i.e., cell substates, e.g., CD4+ memory T cells, naive B cells, etc.), which were classified by comparison with reference datasets (25). All the major mononuclear immune cell lineages, divided into a total of 17 cell substates, were identified from cells isolated using either Ficoll or Cryo-PRO, each clustered independently but projected onto a shared set of two-dimensional uniform manifold approximation and projection (UMAP) axes for visualization (Figure 3a; see Methods). Our analysis identified the MS1 monocyte state that we previously discovered in cohorts of patients with sepsis (10). The average expression of key identifying genes (Figure 3b, color scale) and cell surface proteins (Figure 3c, color scale) was similar between Ficoll and Cryo-PRO methods for each cell type and substate, as was the proportion of cells for which these features could be detected (Figure 3b-c, dot size).

Top marker genes to distinguish each cell type and substate were identified using the FindMarkers function in Seurat (21); rank was determined by fold-change of the gene expression within cells of each cluster compared to the cells outside of the cluster (26). Of the top 30 marker genes for each cell type, shared genes between processing methods ranged from 24 to 28, and shared genes between processing methods for cell substates ranged from 14 to 29 (Supplemental Table 1). Notably we observed a high degree of overlap of top MS1 marker genes between processing methods, with 21 of the top 30 marker genes in common (Supplemental Table 1c-d) and similar expression patterns of key MS1 marker genes (Supplemental Figure 3a).

In an orthogonal approach, we used FindMarkers and the DESeq2 package in R to compare gene expression between all cells processed by the two methods to identify differentially expressed genes (Supplemental Table 2). Of the statistically significant (p<0.05) genes, we did not observe substantial (greater than 4) fold-change differences in expression between the two methods. Most genes with more than a 2-fold expression change were non-coding genes, with the exceptions of the genes CXCL8, FOSB, and JUN being slightly up-regulated in Ficoll cells (Figure 3d). Similar differentially-expressed genes were identified when comparisons were performed within each major cell type (Supplemental Figure 3b), instead of all cells combined. Crucially, no genes that are used to identify cell lineages or cell types were differentially expressed by more than a 2-fold change. Pathway analysis could not be performed due to the sparse number of substantially differentially expressed genes. However, immediate early genes are a class of genes commonly transiently upregulated in many types of cells as a primary response to a variety of stimuli; the presence of the immediate early genes JUN and FOSB may suggest an early response to *ex vivo* stimulation in Ficoll cells (27, 28).

### Cryo-PRO and Ficoll yield similar immune cell type and substate abundances

Defining the composition of circulating immune cells and their substates on a per-patient level is an informative application of scRNA-seq. In sepsis, heterogeneity in the distribution of immune cell types and states between patients is thought to contribute to differences in illness trajectory, outcomes, and response to therapies (29). To evaluate the congruence between methods for characterizing immune cell profiles in sepsis, we computed proportions for each cell type and substate for each sample processed using both Ficoll and Cryo-PRO methods. We defined proportion of cell type as the number of cells of a particular type (e.g., B cells) divided by the number of all PBMCs combined. For cell substates, proportion was defined as the number of cells assigned to a substate divided by the total number of cells of that cell type (e.g., number of CD16+ monocytes divided by the total number of monocytes). We compared cell type proportions between paired Ficoll and Cryo-PRO samples from each of the 24 subjects by investigating their correlations. Substate proportions were calculated for substates of monocytes, T cells, B cells and dendritic cells. Natural killer cells did not demonstrate unique substates; therefore substate proportions were not computed.

**Figure 4.**
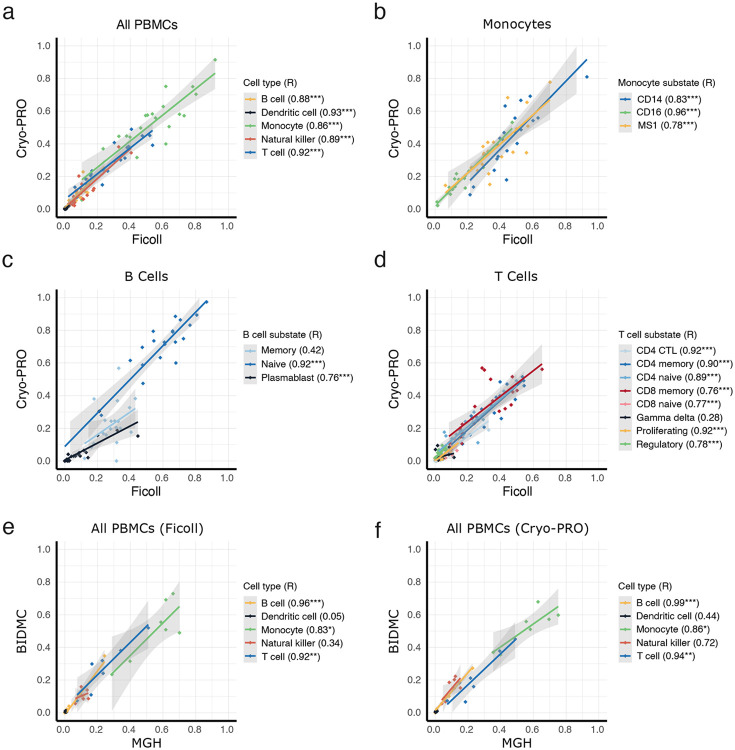
Trends in cell type and substate proportion by patient between method and processing center. **(a)** Scatter plot of cell type proportion from Ficoll and Cryo-PRO. Each point represents the proportion of one cell type from one patient sample, as measured by each method. Each cell type is represented by a different color and trendline. Proportion is the number of cells of one cell type divided by the total number of PBMCs from that patient sample. Patient-paired Ficoll:Cryo-PRO samples are plotted to assess correlation by method for each patient. Pearson’s correlations (R) are shown for all correlations (*p < 0.05, ** p < 0.01, ***p < 0.001). **(b-d)** Scatter plots of cell substate proportion from Ficoll and Cryo-PRO. Each point represents the proportion of one cell substate from one patient sample, as measured by each method. Each cell substate is represented by a different color and trendline. Proportion is the number of cells of one cell substate divided by the total number of cells from its cell type from that patient sample. Patient-paired Ficoll:Cryo-PRO samples are plotted to assess correlation in method for each patient. **(e-f)** Scatter plots of cell type proportion from different processing sites. Each point represents the proportion of one cell type from one patient sample, processed at each site. Each cell type is represented by a different color and trendline. Proportion is the number of cells of one cell type divided by the total number of PBMCs from that patient sample. The patient-paired Ficoll:Ficoll samples and Cryo-PRO:Cryo-PRO samples from the two different enrollment sites are plotted to assess variation in technical duplicates for each patient.

Proportions of cell types were significantly correlated between methods (Figure 4a), with Pearson correlations (R) ranging between 0.86 and 0.93 (p<0.001 for all comparisons). There were significant positive correlations between methods for most substates (R values from 0.48 to 0.96; p<0.05); the exceptions were memory B cells (R = 0.42; NS, not significant) and gamma delta T cells (R = 0.28, NS) (monocytes, B cells, and T cells: Figures 4b-d, dendritic cells: Supplemental Figure 4). Within monocytes, correlations across methods were higher for CD16+ monocytes (R = 0.96, p<0.001) than for CD14+ MS1 (R = 0.78, p<0.001) and classical CD14+ (R=0.83, p<0.001). The poorly correlated memory B cell proportions may be influenced by the fact that naive and memory B cells exist on a continuum (30) such that stochastic differences in clustering may have a bigger impact in assignment between these similar substates. Correlations for the CD14+ monocyte substates (MS1 versus classical) may have been affected by transcriptional similarity as well. Gamma delta T cells were present in very low numbers across each method, with enhanced effects of outliers likely impacting correlations. Comparison of cell substate proportions between Ficoll and Cryo-PRO at an individual subject level showed a high degree of similarity across each of the 24 subjects analyzed (Supplemental Figure 5), especially for the 15 patients whose samples were processed immediately at clinical sites (Supplemental Figure 5a).

To evaluate the robustness of our Cryo-PRO approach, we assessed the technical reproducibility of scRNA-seq results for the same blood samples processed at different clinical sites. We compared cell type and substate abundances for the 8 patients whose samples were processed simultaneously at MGH and BIDMC, noting inherent processing delays due to sample transport between sites (see Methods). Proportions of major cell types (monocytes, B cells, and T cells) were highly correlated when the patient sample was simultaneously processed at different clinical sites for Ficoll (R values from 0.83 to 0.96, p<0.001) (Figure 4e) and Cryo-PRO (R values from 0.86 to 0.99, p<0.001) (Figure 4f). Dendritic cells (Ficoll R = 0.05, Cryo-PRO R = 0.44; both NS) and natural killer cells (Ficoll R = 0.34, Cryo-PRO R = 0.72; both NS) were poorly correlated between sites, possibly due to small overall cell counts and variable yield between processing runs that exaggerate differences in cell proportions. For cell substates, correlations were significant for nearly all substates of monocytes, T cells, and B cells, and dendritic cells for each method between sites (Supplemental Figure 6), though for some substates including MS1, cross-site correlations were slightly lower for Cryo-PRO (right column) than Ficoll (left column).

## DISCUSSION

Single-cell transcriptional profiling facilitates high-resolution characterization of the heterogeneity among circulating immune cells, thereby revealing critical insights into diseases such as sepsis where the immune response plays a pivotal role (31). Performing these investigations with clinical samples is critical for translational goals, as it establishes a direct link between cellular transcriptomics and patient-derived data. However, the current state-of-the-art process for scRNA-seq faces a number of major roadblocks to application on clinical samples: intensive sample collection strategies that require more time, equipment, and molecular techniques than are typically available to clinical study teams; and cost. ScRNA-seq is becoming more economical with emerging technologies and the ability to pool samples, but performing scRNA-seq from patient blood still requires PBMC isolation via the time- and resource-intensive process of Ficoll density gradient centrifugation. This limitation has greatly constrained the application of scRNA-seq in clinical investigations, resulting in smaller clinical cohorts that may not fully capture the heterogeneity of diseases under study.

Here, we demonstrated that direct cryopreservation of a small volume (~1 mL) of whole blood at the point of care, followed by thawing and PBMC isolation at a centralized research facility, is a viable alternative to on-site Ficoll processing for scRNA-seq and CITE-seq. This simple and streamlined approach significantly reduced the time and technical expertise needed to obtain clinical samples, while still preserving single-cell transcriptomes and surface proteomes in patients with sepsis. We independently identified the same immune cell types and substates in the datasets of Cryo-PRO and Ficoll, including the sepsis-enriched monocyte substate MS1, considered important in sepsis immunopathophysiology (10, 32). We found a high correlation between methods for the abundances of all major cell types. Although cell substates may be less distinctly defined by their transcriptional profile and are therefore more susceptible to misidentification due to stochasticity in clustering, we still observed high correlations between most substate proportions derived from the two methods after independent clustering and substate assignment. Moreover, we observed similar patterns of gene and surface protein expression across cell types and substates with very minimal differential gene expression between methods. Together, this substantial equivalence between the gold-standard method of Ficoll processing and Cryo-PRO demonstrates that Cryo-PRO does not introduce major artifacts from processing and generates results with biological significance in patients. When deployed across two different enrolling emergency departments, cell type and substate abundances from Cryo-PRO showed strong correlations across sites. This finding shows that Cryo-PRO is robust to variations in collection site and operator, further validating it as a reliable strategy for expanding scRNA-seq studies.

The Cryo-PRO method has transformative potential for multicenter sample collection and clinical trial enrollment efforts by greatly simplifying on-site protocols for scRNA-seq and transferring the technically demanding steps to a centralized location. The resource demands of onsite processing for scRNA-seq particularly impact studies of highly heterogeneous diseases with acute onset where study collection strategies must be deployable at any time a patient may present. Sepsis is an archetype of such a condition, and sample sizes for scRNA-seq studies of sepsis have, as a consequence, been too small to bring the full power of the method to bear on investigating biological reasons underlying the clinical heterogeneity of the condition (10, 13-16). The substantial reduction in technical skill and time requirements for sample processing and preservation (i.e., mean time of 13 minutes for Cryo-PRO vs 143 minutes for Ficoll) has crucial operational implications in the clinical research setting. Simplifying sample collection also offers an opportunity for improving cost efficiency by enabling the rapid enrollment of many potentially suitable patients for clinical studies, followed by retrospective adjudication based on subsequent clinical course to inform the selection of appropriate patients for sequencing. Widening the net of subjects enrolled in this manner would better reflect the true patient heterogeneity in conditions under study, enabling post-hoc enrichment for rare clinical phenotypes or outcomes. For sepsis, this strategy could facilitate the derivation of scRNA-seq-based endotypes on a large, diverse cohort of sepsis patients with varied clinical presentations and demographic backgrounds, including those from hospitals in underserved communities without dedicated research teams and resources to typically participate in clinical research.

Other forms of rapid whole blood cryopreservation have recently been demonstrated with scRNA-seq (33, 34). In one of these studies, a substantial loss in the fractional abundance of myeloid cells was observed when compared with samples obtained using Ficoll (33). Our approach produces better equivalence with the standard Ficoll method across immune cell types. Another method (34) is based on the use of fixed cells, which provides more flexibility in the cryopreservation process compared to Ficoll (35). However, because fixation impairs polymerases involved in cDNA library preparation, fixed cell RNA profiling requires hybridization to a predefined set of probes, rather than sequencing, to detect transcripts (36), introducing a number of limitations. In particular, hybridization-based approaches require *a priori* knowledge of the cell’s potential transcriptional signature, and thus fail to capture regions of high allelic diversity such as BCR and TCR clonotypes. Additionally, sample processing and preservation with fixed cell profiling is generally kit-specific, requiring users to commit to a technology prior to the start of sample collection and use the costly reagents for all collected samples. In addition, these prior approaches, in part due to smaller sample size, relied on co-clustering of scRNA-seq data with the traditional Ficoll method to assign cell states. In order to be useful at the point of care, any streamlined collection method must stand alone; we therefore independently clustered and analyzed patient-matched data from Cryo-PRO alone, versus Ficoll alone, and found substantial technical and biological equivalence.

Our study has several limitations. First, our cohort size remains small in the context of sepsis studies. However, to the best of our knowledge, this study (n = 24 subjects and 32 paired samples) is the largest to date evaluating the feasibility of whole blood cryopreservation for scRNA-seq and CITE-seq in any context, and demonstrates substantial equivalence with conventional methods. We aim to further validate Cryo-PRO as a sample processing approach in a larger cohort of subjects in the future. Second, although all major cell types and substates had substantial equivalence in patient-level abundance, some cell substate abundances deviated between Cryo-PRO and Ficoll methods. Some differences in substate assignment within cell types (e.g., MS1 versus classical CD14+ monocytes or memory versus naive B cells) are less well-defined than differences in cell types, and may reflect more of a continuum than a dichotomy so more stochastic differences in assignments are expected. Other cell substates like gamma delta T cells and substates of dendritic cells were present at very low abundances and therefore more susceptible to outlier effects. While some differences between methods may reflect differences in either gene expression or survival by cell type and substate, each method introduces processing steps that may perturb transcription, i.e., centrifugation for 2 hours through a density gradient followed by freezing, thawing, and flow cytometry for Ficoll; exposure to DMSO, freezing, thawing, magnetic cell separation, and flow cytometry for Cryo-PRO. Additionally, 8 of our 23 subjects were processed in parallel at two sites, with a resulting mean delay of just over 2 hours prior to processing by either method, which may have affected subsequent sequencing results in these samples. Despite these limitations, the overall agreement in cell type and substate proportions between methods, and the minimal perturbations seen in differential expression by method suggests that major transcriptional signals that reflect relevant biology are preserved. Third, this study does not yet assess the function of PBMCs isolated by Cryo-PRO, whereas Ficoll preparation is known to yield functional PBMCs (35), enabling correlation of transcriptional states with cellular activity. We plan future studies to assess the functional capacity of PBMCs isolated using the Cryo-PRO method.

By simplifying sample collection at the point of care, Cryo-PRO can unlock greater potential of scRNA-seq to study the biology of complex clinical conditions across multiple collection sites, including lower-resource settings, thus enabling better capture of the true heterogeneity of diseases. This method greatly lowers the barrier to embedding scRNA-seq-compatible collection strategies in randomized clinical trials, which would enable post-hoc analyses to identify biological subsets of patients (i.e., endotypes) that may selectively respond to therapeutic interventions. In addition, Cryo-PRO could enhance the cost-efficiency of scRNA-seq by enabling “overcollection” at the point of care, reserving PBMC isolation and scRNA-seq only for samples from patients who display clinical phenotypes or disease trajectories of interest on subsequent adjudication. Thus, Cryo-PRO has substantial potential to expand the application of scRNA-seq towards personalized medicine in complex and heterogeneous conditions like sepsis, and this work represents an important first step towards that goal.

## METHODS

### Patient enrollment and clinical adjudication.

This study was approved by the Massachusetts General Brigham IRB (2022P002833) and conducted at Massachusetts General Hospital and Beth Israel Deaconess Medical Center. Inclusion criteria were adult patients arriving to the Emergency Department with evidence of organ dysfunction for whom bacterial infection was possible or suspected. Eligible patients had a blood sample collected under an IRB-approved alteration of informed consent, which allowed a research sample to be drawn simultaneously with the initial clinical blood draw. Informed consent was obtained from the patient or a surrogate at a later time after initial resuscitation.

Sample was collected for 100 patients during a 12-month period from April 2023 to March 2024. Of those 100, consent to analyze sample for research was obtained in 84 patients, thus considered enrolled. Sample was discarded for those who did not provide consent. Clinical data were collected on all enrolled subjects and entered into REDCap by clinical research coordinators. Physician adjudication (MRF) was later performed via retrospective chart review with access to all available clinical data and notes during the subject’s hospitalization. Subjects were adjudicated as meeting Sepsis-3 criteria (19) for sepsis or septic shock during the first 48 hours of hospitalization, or whether infection without sepsis versus other non-infectious cause for presenting illness was present. For the current analysis, we prioritized sequencing in those subjects adjudicated as sepsis and septic shock. We selected 23 subjects to be sequenced and included in the analysis.

### Sample collection at clinical sites.

Research blood samples were collected in 10 mL EDTA tubes. Up to 20 mL was collected if patient samples were being parallel-processed at both clinical sites; up to 10 mL was collected if patient samples were being processed at only a single site. For samples parallel-processed at both clinical sites, one of the two 10 mL EDTA tubes collected at the enrolling site was couriered to the second site.This resulted in a delay in processing of about 2 hours on average; samples from one subject were delayed >3 hours. Samples obtained for single-site processing were taken directly to the onsite lab for immediate processing. Processing of all 10 mL EDTA samples involved cryopreservation of whole blood (2 mL) and onsite density gradient centrifugation with Ficoll (~3 to 6 mL) as described below. Up to 3 mL of the collected whole blood sample was used for other research purposes.

### Onsite whole blood cryopreservation for Cryo-PRO.

For immediate whole blood cryopreservation, 2.0 mL of blood from the 10 mL EDTA tube were mixed with 200 uL DMSO. Two 1-mL aliquots in cryovials were then prepared per sample and were slowly cooled using a Mr Frosty (Sigma-Aldrich) in a −80°C freezer. Aliquots were stored onsite at −80°C for less than 1 month before being transported to the Broad Institute (Cambridge, MA) on dry ice and immediately stored at −140°C until the time of sequencing. Two 1 mL aliquots were cryopreserved in order to have a backup sample if needed.

### Onsite density gradient centrifugation (Ficoll) and cryopreservation of PBMCs.

Density gradient centrifugation was performed on the remaining blood in the EDTA tubes (~3 to 6 mL). Blood with EDTA was diluted 1:1 with room temperature PBS and layered over Ficoll-Paque PLUS density gradient media (Cytivia) in a SepMate tube (STEMCELL) before centrifuging at 1,200 rcf for 20 minutes at 20°C with slow acceleration and the brake off. The buffy coat layer was carefully collected and washed twice with cold RPMI (Gibco) before cells were counted, resuspended in CryoStor CS10 (STEMCELL), and aliquoted into cryotubes targeting 1 million cells per vial. Samples were cooled, stored, and transported in the same manner as the Cryo-PRO samples.

### Healthy donor blood cryostorage.

Fresh healthy donor blood in EDTA tubes was ordered from Research Blood Components (Watertown, MA) and processed within two hours of receipt. Whole blood Cryo-PRO and Ficoll PBMC cryostorage steps took place as described, though all processing steps occurred at the Broad Institute.

### Pre-sequencing processing for both Ficoll and Cryo-PRO samples.

All subsequent processing and analysis steps described here were performed at the Broad Institute. On the day of flow cytometry sorting and Chromium 10X processing, a sample of cryopreserved whole blood (for Cryo-PRO) and a Ficoll sample were thawed for each patient. Sequencing batches were designed to contain four Ficoll samples and four patient-matched Cryo-PRO samples to minimize the effect of sequencing batch variation on the method comparison; therefore, 8 samples total were processed in parallel.

For each of the four Cryo-PRO samples, 1 mL of cryopreserved whole blood was thawed in a 37°C water bath for 1 min 15 seconds and transferred into a 5-mL polystyrene round-bottom tube using 1 mL of PBS containing 2 mM EDTA and 2.5% FBS. Samples were immediately depleted of red blood cells using the STEMCELL EasySep RBC depletion kit. Briefly, the diluted blood was mixed with 50 uL of the RBC depletion reagent before immediately being placed on a magnet for 5 minutes at room temperature. The supernatant was pipetted off and mixed with an additional 50 uL of RBC depletion reagent in a new tube before another immediate 5 minute magnet incubation. At the end of the second incubation, the supernatant was transferred into 8.5 mL of FBS-RPMI (RPMI + 10% FBS + 1x penicillin/streptomycin) on ice. These steps were performed in parallel for the four Cryo-PRO samples.

For each of the four Ficoll samples, one vial per patient was thawed in a 37°C water bath for 1 min 15 seconds before transfer with 1 mL of FBS-RPMI into 8.5 mL of FBS-RPMI on ice. For patients with three or more Ficoll vials, two vials were thawed and combined to improve cell recovery. These steps were performed in parallel for the four Ficoll samples.

For the subsequent steps, Cryo-PRO and Ficoll samples received the same treatment and steps were performed in parallel. All samples were centrifuged to pellet the cells (300xg, 5 minutes, 4°C), then resuspended with FACS-PBS (PBS + 2mM EDTA + 2.5% FBS) and centrifuged again. Each sample was then resuspended in 50 uL FACS-PBS and incubated on ice with a hashtag oligo for pooled sequencing (TotalSeq^™^ anti-human Hashtags, BioLegend), an Fc receptor blocking solution (Human TruStain FcX^™^, BioLegend), and flow cytometry stains (DAPI solution,Thermo Scientific; Alexa Fluor^®^ 700 anti-human CD15 [Clone: HI98], BioLegend; FITC anti-human CD235a [Clone: HI264], BioLegend; and PE anti-human CD45 [Clone: HI30], BioLegend). Samples were then washed in cold FACS-PBS and sorted on a SONY MA800 cell sorter to select for DAPI− CD15− CD235a− CD45+ cells, with a sorting target of 50,000 cells per sample.

After sorting, the hashed and sorted cells from all eight samples were pooled, pelleted (300xg, 5 minutes, 4°C in FACS-PBS), and resuspended in a CITE-Seq cocktail for surface proteome measurement (20) for a final incubation on ice. After 20 minutes, the cells were washed twice more (centrifugation at 300xg, 5 minutes, 4°C followed by resuspension in PBS + 2.5% FBS), counted, and resuspended in PBS + 2.5% FBS for a target concentration of 1,000 cells/uL.

### Library construction and scRNA sequencing.

Droplet-based single-cell RNA capture and RNA and ADT library construction was performed with the Chromium single-cell 5’ kit v2 (10x Genomics, Inc). Forty uL of cells were loaded onto the Chromium Chip K, and Gel Bead-in Emulsion creation and library construction followed according to the manufacturer’s protocol (37). Eight batches of libraries were prepared (including gene expression libraries and cell surface protein libraries), with each batch barcoded using the 10X Dual Index Kit and sequenced altogether. Gene expression libraries were sequenced at a low depth (~200 reads/cell) using the Illumina MiniSeq 150 Cycle Hi-Output Kit for a quality check and cell count estimate to inform library balancing. Rebalanced libraries targeting 50,000 reads/cell for gene expression and 10,000 reads/cell for surface proteins were then sequenced on an Illumina NovaSeq S4.

### Data preprocessing.

FASTQ files were aligned to a reference genome (GRCh38) using the Cell Ranger v6 pipeline by 10X Genomics. Demultiplexing and multiplet detection with patient hashtag oligos was performed using the Cumulus pipeline (36). Filtered gene expression matrices and CITE-Seq matrices were then analyzed using the Seurat V5 package in R. Multiplets and cell barcodes without corresponding gene expression, CITE-seq, and demultiplexing data were removed. Genes present in less than 10 cells were removed. Sequencing data from each method was split into two datasets and analyzed independently. For each set, RNA expression data was normalized, scaled, and integrated between sequencing batches using the top 2,000 most variable genes. Scaled CITE-seq data was integrated by finding multimodal neighbors using the first 50 principal components of RNA and CITE-seq data.

### Clustering and substate identification.

Clustering was performed using the resulting weighted-nearest-neighbors graph, and the Clustree package (38) was used to determine clustering resolution. Cell types were assigned to clusters using top marker genes for each cluster (determined by Wilcoxon rank-sum test, Bonferroni-corrected p-value < 0.05, ranked by fold-change), and cell substates were assigned using top marker genes obtained by subsetting and re-clustering cells from each cell type at a higher resolution. Classification of cell types and substates was cross-referenced using the annotated Azimuth reference dataset (25). Clusters were defined as low quality if over 20% of cells in the cluster were cells with mitochondrial genes representing 10% or more of total genes detected in that cell. Low quality clusters were removed from further analysis as part of an extended quality control. After method-independent cell substate assignment, the Ficoll and Cryo-PRO datasets were combined and a UMAP was generated using the weighted-nearest-neighbors graph for the purpose of data visualization.

### Differential gene expression.

To assess differential gene expression between methods, scRNA-seq data was first pseudo-bulked by sample (generating 32 “bulk” RNA-seq samples from each method) to minimize p-value inflation (39), and FindMarkers with the DESeq2 package was used to detect differentially expressed genes. The same process occurred for differential gene expression at the cell type level, although cells were first pseudo-bulked by cell type in addition to sample.

Top marker genes for each cell substate were calculated in Seurat using the FindMarkers function, and genes with an expression log2 fold-change > 0.25, genes expressed in over 25% of cells in the cluster; and a Bonferroni-corrected p-value < 0.05 were included.

### Cell type and substate abundance.

PBMC cell type proportions were calculated as a fraction of all major cell types identified (monocytes, B cells, T cells, NK cells, and dendritic cells). Cell substate proportions were calculated as a fraction of the cell type in question. Cell clusters defined as low quality, or belonging to a class of cells other than PBMCs (i.e., platelets and hematopoietic stem and progenitor cells), were not included in proportion calculations. Samples with fewer than 1,000 total cells were not included in correlation calculations, in Figure 4 or in Supplemental Figures 4 and 6 due to effects of low sample sizes. When possible, the Ficoll:Cryo-PRO comparisons were made using samples processed at the same site in which they were collected. In the case of Subject 17, one sample yielded less than 1,000 cells, so the Ficoll:CrypPRO samples processed at the alternative site were used in calculations instead. R values were calculated for the scatterplots of cell types and substates shown using a Pearson correlation.

### Figure generation.

Figure 1 was created in BioRender (40). Subsequent figures were generated using the ggplot2 package, the ScCustomize package (41), and the Seurat package in R (25).

## Supplementary Material

Supplement 1 **Supplemental Table 1.** Top marker genes by cell cluster and method. **(a)** Top 30 marker genes (ordered by highest average log_2_FC) for each cell type by method. **(b)** Shared top marker genes for each cell type (genes in (a) appearing across both methods). **(c)** Top 30 marker genes (ordered by highest average log_2_FC) for each cell substate by method. **(d)** Shared top marker genes for each cell substate (genes in (c) appearing across both methods). Marker genes were excluded from the list if expressed in fewer than 25% of cells in that cluster (see Methods).
**Supplemental Table 2.** Differential gene expression by method. List of genes that were significantly (adjusted p value < 0.05) differentially expressed between methods for cell types and across all cells. Positive average log_2_FC indicates that the gene was enriched in Ficoll cells; negative average log_2_FC indicates that the gene was enriched in Cryo-PRO cells. Calculations were performed on pseudo-bulked samples (see Methods).

Supplement 2 **Supplemental Figure 1.** Number of singlet cells sequenced per method. Starting blood sample volume was variable in Ficoll samples and was 1 mL in Cryo-PRO samples.
**Supplemental Figure 2.** Per-sample violin plots showing UMIs of RNA transcripts **(a)**, unique genes **(b)**, percentage of mitochondrial transcripts **(c)**, unique surface protein features detected via CITE-seq **(d)**, and UMIs of surface protein features detected via CITE-seq **(e)** per cell. Batches represent samples that were thawed, processed and sequenced together. Ficoll and Cryo-PRO samples from the same patient are plotted next to each other. For patients where parallel processing occurred at both clinical sites (bottom rows), the samples processed at the opposite site of enrollment are shown in lighter shades. A total of 137 different surface proteins were queried in the CITE-seq analysis. PRO denotes Cryo-PRO.
**Supplemental Figure 3.** **(a)** Dot plots of marker gene expression by each monocyte substate. Color represents scaled relative expression (blue = higher expression). Size represents the proportion of cells in each substate where the feature was detected. **(b)** Volcano plots showing genes differentially up-regulated (positive Log_2_FC) or down-regulated (negative Log_2_FC) in Ficoll compared to Cryo-PRO after pseudobulk analysis. Genes with adjusted p-values of less than 0.05 are shown in red; those with p < 0.05 and abs(log_2_FC) > 1 are labeled. Plots are shown for differential gene expression among all cells (top left) and for each major cell type (subsequent plots).
**Supplemental Figure 4.** Scatter plot of dendritic cell substate proportion from Ficoll and Cryo-PRO. Each point represents the proportion of one cell substate from one patient sample, as measured by each method. Each cell substate is represented by a different color and trendline. Proportion is the number of cells of one cell substate divided by the total number of dendritic cells from that patient sample. Patient-paired Ficoll:Cryo-PRO samples are plotted to assess correlation in method for each patient. Pearson’s correlations (R) are shown for all correlations (*p < 0.05, ** p < 0.01, ***p < 0.001).
**Supplemental Figure 5.** Cell substate proportions for technical duplicate samples processed at single centers **(a)** and technical duplicate samples processed at both centers **(b)**. Samples from the same patient processed using different methods are shown next to each other; in (b), the corresponding pair of technical duplicates are shown subsequently. PRO denotes Cryo-PRO.
**Supplemental Figure 6.** Scatter plots of cell substate proportions from different processing sites. Each cell substate is represented by a different color and trendline. Proportion is the number of cells of one cell substate divided by the total number of cells from its cell type from that patient sample. The patient-paired Ficoll:Ficoll samples and Cryo-PRO:Cryo-PRO samples from the two different enrollment sites are plotted to assess correlation of technical duplicates for each patient. Pearson’s correlations (R, *p < 0.05, ** p < 0.01, ***p < 0.001) are shown for all correlations.
